# Mitochondrial Dynamics and VMP1-Related Selective Mitophagy in Experimental Acute Pancreatitis

**DOI:** 10.3389/fcell.2021.640094

**Published:** 2021-03-18

**Authors:** Virginia Vanasco, Alejandro Ropolo, Daniel Grasso, Diego S. Ojeda, María Noé García, Tamara A. Vico, Tamara Orquera, Jorge Quarleri, Silvia Alvarez, María I. Vaccaro

**Affiliations:** ^1^Universidad de Buenos Aires, Consejo Nacional de Investigaciones Científicas y Técnicas (CONICET), Instituto de Bioquímica y Medicina Molecular (IBIMOL), Facultad de Farmacia y Bioquímica, Buenos Aires, Argentina; ^2^Universidad de Buenos Aires, Consejo Nacional de Investigaciones Científicas y Técnicas (CONICET), Instituto de Investigaciones Biomédicas en Retrovirus y SIDA (INBIRS), Facultad de Medicina, Buenos Aires, Argentina

**Keywords:** pancreatitis, autophagy, mitophagy, VMP1, mitochondrial dynamics, mitochondrial function, Parkin1, DRP1

## Abstract

Mitophagy and zymophagy are selective autophagy pathways early induced in acute pancreatitis that may explain the mild, auto limited, and more frequent clinical presentation of this disease. Adequate mitochondrial bioenergetics is necessary for cellular restoration mechanisms that are triggered during the mild disease. However, mitochondria and zymogen contents are direct targets of damage in acute pancreatitis. Cellular survival depends on the recovering possibility of mitochondrial function and efficient clearance of damaged mitochondria. This work aimed to analyze mitochondrial dynamics and function during selective autophagy in pancreatic acinar cells during mild experimental pancreatitis in rats. Also, using a cell model under the hyperstimulation of the G-coupled receptor for CCK (CCK-R), we aimed to investigate the mechanisms involved in these processes in the context of zymophagy. We found that during acute pancreatitis, mitochondrial O_2_ consumption and ATP production significantly decreased early after induction of acute pancreatitis, with a consequent decrease in the ATP/O ratio. Mitochondrial dysfunction was accompanied by changes in mitochondrial dynamics evidenced by optic atrophy 1 (OPA-1) and dynamin-related protein 1 (DRP-1) differential expression and ultrastructural features of mitochondrial fission, mitochondrial elongation, and mitophagy during the acute phase of experimental mild pancreatitis in rats. Mitophagy was also evaluated by confocal assay after transfection with the pMITO-RFP-GFP plasmid that specifically labels autophagic degradation of mitochondria and the expression and redistribution of the ubiquitin ligase Parkin1. Moreover, we report for the first time that vacuole membrane protein-1 (VMP1) is involved and required in the mitophagy process during acute pancreatitis, observable not only by repositioning around specific mitochondrial populations, but also by detection of mitochondria in autophagosomes specifically isolated with anti-VMP1 antibodies as well. Also, VMP1 downregulation avoided mitochondrial degradation confirming that VMP1 expression is required for mitophagy during acute pancreatitis. In conclusion, we identified a novel DRP1-Parkin1-VMP1 selective autophagy pathway, which mediates the selective degradation of damaged mitochondria by mitophagy in acute pancreatitis. The understanding of the molecular mechanisms involved to restore mitochondrial function, such as mitochondrial dynamics and mitophagy, could be relevant in the development of novel therapeutic strategies in acute pancreatitis.

## Highlights

- The novel DRP1-Parkin1-VMP1 autophagy pathway, which mediates the selective degradation of damaged mitochondria by mitophagy, was unraveled in acute pancreatitis.
- Mitochondrial O_2_ consumption and ATP production significantly decreased early after induction of acute pancreatitis, with a consequent decrease in the ATP/O ratio.
- Mitochondrial fission, mitochondrial elongation, and mitophagy are rapidly activated in pancreatic acinar cells during experimental mild pancreatitis in rats.

## Introduction

Acute pancreatitis (AP) is a pancreatic inflammatory condition whose global estimates of incidence and mortality are between 33 and 74 cases per 100,000 person-years, with 1–60 deaths per 100,000 person-years for AP (Xiao et al., 2016). Most patients develop a mild and auto limited form of the disease, but about 20% of them suffer a severe disease presenting pancreatic necrosis, which can spread within the same pancreatic tissue and be accompanied by injury to other organs. Moreover, within deaths associated with AP, half of them occur during the first week due to multiple organ failure (Mukherjee et al., 2008). Despite recent advances, the pathogenesis of cellular damage occurring during inflammation in the severe form of the disease is unclear and no adequate specific treatments have been developed (Maléth et al., 2013).

Previous studies of our laboratory identified VMP1 (NM_138839) as a novel autophagy-related protein in which its expression is induced in the human pancreas with pancreatitis and in experimental pancreatitis under the G-coupled receptor CCK-R hyperstimulation (Ropolo et al., 2007; Vaccaro et al., 2008; Grasso et al., 2011). We also described the selective autophagic pathway, zymophagy, as an early protective mechanism in AP preventing acinar cell death (Grasso et al., 2011). Zymophagy is a selective type of autophagy that occurs during AP. It may be induced by CCK-R hyperstimulation, mediated by VMP1 expression, which recognizes and sequesters those zymogen granules that are initially activated by the disease. We propose that zymophagy is a protective mechanism set up by the acinar cell, and that may account for the self-limited form of AP (Vaccaro, 2012).

The relevance of autophagy as a protective mechanism in pancreatitis has been further confirmed in mice using pancreas-specific autophagy-related proteins (ATG) ATG5 or ATG7 knockout mice. These animals developed severe pancreatitis, with trypsinogen activation, fibrosis, inflammation, acinar-to-ductal metaplasia, and pancreas atrophy (Antonucci et al., 2015; Diakopoulos et al., 2015). In this context, mitochondria are essential players, not only as the main source of ATP and other biomolecules required during autophagy processes, but also in the regulation of intracellular Ca^2+^ homeostasis in the acinar cells. Therefore, a normal mitochondrial function could be necessary for zymophagy in response to AP. In this sense, it was described that mitochondria-deficient cells exhibit attenuated autophagic gene induction and autophagic flux in response to starvation (Graef and Nunnari, 2011). Even more, mitochondria are direct targets of damage during AP, since fatty acid ethyl esters (FAEEs) that have been reported increased in AP patients and in several models of AP tend to accumulate in the inner mitochondrial membrane affecting the mitochondrial polarization, producing uncoupling of oxidative phosphorylation, and inhibiting ATP production (Mukherjee et al., 2008). Therefore, cellular survival depends on the recovered mitochondrial function and efficient clearance of dysfunctional mitochondria.

The mitochondrial population is highly dynamic within cells, mitochondria form networks continually undergo fusion and fission events during cell life (Bereiter-Hahn and Voth, 1994). In mammals, mitochondrial fusion is mainly regulated by OPA1 proteins (optic atrophy 1, an internal mitochondrial membrane GTPase) (Cipolat et al., 2004), and by outer membrane GTPases or Mitofusin 1 and 2 (Mnf) (Santel and Fuller, 2001). Mitochondrial fission is mainly controlled by the cytosolic GTPase protein DRP1 (dynamin-related protein 1), whose translocation from cytoplasm to mitochondria is an essential step at the beginning of mitochondrial fragmentation (Smirnova et al., 2001). It is postulated that mitochondrial dynamics respond to cellular energy requirements (Liesa and Shirihai, 2013) triggered by other intracellular processes such as redox changes and autophagy (Willems et al., 2015; Cid-Castro et al., 2018; Ježek et al., 2018). Mitophagy, a selective form of autophagy, is a major route for the removal of damaged mitochondria. During mitophagy, mitochondria are sequestered in double-membrane vesicles and delivered to lysosomes for degradation. Narendra et al. (2008, 2010) found that the decrease of mitochondrial membrane potential leads to PTEN-induced kinase 1 (PINK1) accumulation in the mitochondrial outer membrane, leading to the ubiquitination of damaged mitochondria by Parkin1, consequently carrying out autophagic degradation of dysfunctional mitochondria (Vincow et al., 2013). Therefore, it is important to unravel the mechanisms involved in the recovery of mitochondrial function that might be relevant in cellular and tissue recovery processes during mild AP.

Mitochondrial dysfunction has been reported in various models of severe AP in which loss of mitochondrial membrane potential and mitochondrial fragmentation seems to be involved (Shalbueva et al., 2013; Mukherjee et al., 2016; Biczo et al., 2018). Regardless of the underlying mechanisms, genetic or pharmacological prevention of mitochondrial depolarization resulted in the restoration of mitochondrial function, with a large decrease in local and systemic pathological responses in models of experimental severe pancreatitis (Shalbueva et al., 2013; Mukherjee et al., 2016; Biczo et al., 2018). However, the relevance of mitochondria function and dynamics in experimental models of mild pancreatitis are poorly elucidated.

In this work, we analyzed mitochondrial function, mitochondrial dynamics, and mitophagy in pancreatic acinar cells during experimental early pancreatitis in rats and in a cell model under the hyperstimulation of the G-coupled receptor for CCK (CCK-R). We have identified a VMP1 mediating pathway in mitophagy, which selectively sequesters and degrades damaged mitochondria during the initial steps of experimental pancreatitis.

## Materials and Methods

### Drugs and Chemicals

Caerulein (CAE) was purchased from Sigma-Aldrich (St. Louis, MO, United States). Rabbit monoclonal antibodies against OPA1 (ab157457, Abcam Plc, Cambridge, United Kingdom), DRP1 (D6C7 rabbit mAb; Cell Signaling), LC3B [LC3B antibody (2775)]; Cell Signaling Technology, MA, United States), p62 [SQSTM1 (P-15): sc-10117; Santa Cruz Biotechnology, Santa Cruz, CA, United States)], VMP1 (D1y3E, Cell Signaling Technology, MA, United States); mouse monoclonal antibodies against Parkin (P6248, Sigma-Aldrich, St. Louis, MO, United States), V5 [13202 V5-Tag (D3H8Q) rabbit mAB-Cell Signaling)], β-actin [β-actin (C4): sc-47778; Santa Cruz Biotechnology, Santa Cruz, CA, United States)], beta tubulin (ab131205, Abcam Plc, Cambridge, United Kingdom); rabbit anti-goat antibody (Santa Cruz Biotechnology, Santa Cruz, CA, United States); goat policlonal antibodies against VDAC-1 (D-16 sc-32063; Santa Cruz Biotechnology, Santa Cruz, CA, United States); rabbit anti-mouse antibody [(315-035-048) Jackson InmunoResearch, Baltimore Pike, United States]; goat anti-rabbit antibody [(GAR):170-5046; Bio-Rad, CA, United States]. Other reagents, enzymes, and chemicals were of reagent grade and also from Sigma-Aldrich.

### Animal Model (*in vivo* Model)

AP was induced through the use of supramaximal dose of CAE, which is a CCK homologue, leading to the activation of the intracellular proteolytic enzyme characteristic of this syndrome (Williams, 2008). Female Sprague-Dawley rats (between 40 to 50 days old) were treated with seven i.p. injections of 50 mg of CAE per kg body weight in 1 h intervals. Treated groups studied: CAE 1, animals sacrificed one hour after the first injection; CAE 3, animals sacrificed after 3 h of treatment; CAE 24 and CAE 48, animals injected and sacrificed at 24 and 48 h, respectively. Control groups (CG) were injected with vehicles following the same scheme as treated groups. Animal experiments were approved by the Animal Care and Research Committee of the School of Pharmacy and Biochemistry, University of Buenos Aires (CICUAL; Exp. 0039150/15), and strictly followed the International Guiding Principles for Biomedical Involving Animals (ICLAS).

### Pancreatic Mitochondria Isolation

Rats were euthanized in CO_2_ chamber and pancreas was excised and placed in ice-cold isolation buffer [58 mM sucrose, 192 mM mannitol, 2 mM Tris/HCl, 0.5 mM EDTA, 0.5% BSA, pH 7.4 (Hodârnâu et al., 1973)]. The tissue was homogenized in 15 ml of isolation buffer with 1 μg/ml pepstatin, 1 μg/ml aprotinin, 1 μg/ml leupeptin, and 0.4 mM PMSF (phenylmethanesulfonyl fluoride) using a glass/Teflon homogenizer. Homogenates were centrifuged at 650 *g* for 10 min, and the sediment which contains nuclei and cell debris was discarded. The supernatant was centrifuged at 8,000 *g* for 10 min to precipitate mitochondria. The mitochondrial pellet was then washed twice, and finally resuspended in the isolation buffer, according to Hordanau modified (Hodârnâu et al., 1973). To assay the purity of isolated mitochondria, the lactate dehydrogenase activity was measured and only mitochondria with less than 5% impurity were used (Cadenas and Boveris, 1980). Protein quantification was performed with the Folin reagent with bovine serum albumin as standard.

### Mitochondrial Oxygen Uptake

Mitochondrial oxygen consumption was measured as described before (Vico et al., 2019), using a Clark-type oxygen electrode for high-resolution respirometry (Hansatech Oxygraph, Norfolk, United Kingdom). Briefly, 0.3–0.4 mg/ml of freshly pancreatic mitochondria were incubated in a respiration medium containing 120 mM KCl, 5 mM KH_2_PO_4_, 1 mM EGTA, 3 mM HEPES, 1 mg/ml BSA, 2 mM malate, and 5 mM glutamate, pH 7.2 at 25°C. Resting respiration rate (state 4) was measured in this condition. In order to measure an active respiration rate (state 3), 1 mM ADP was added (Vanasco et al., 2014). Respiratory control ratio (RCR) was calculated as the ratio between state 3/state 4 respiration rates. Results were expressed as ng-at O/min. mg protein.

### Mitochondrial ATP Production Rate

ATP production rate was measured by the luciferin-luciferase chemiluminescent method in a liquid scintillation counter LKB Wallac 1209 Rackbeta. Freshly pancreatic mitochondria (30–50 μg) were incubated at 28°C in a reaction medium containing 120 mM KCl, 20 mM Tris–HCl, 1.6 mM EDTA, 0.08% BSA, 8 mM K_2_HPO_4_/KH_2_PO_4_, 0.08 mM MgCl_2_, pH 7.4, 40 μM luciferine, 1 μg/ml luciferase. As substrates, 6 mM malate, 6 mM glutamate, and 0.1 mM ADP were added (Vives-Bauza et al., 2007). As control, ATP production rate in the presence of 2 μg/ml oligomycin was determined, and a calibration curve was performed with ATP as standard (0–20 nmoles). Results were expressed as nmol ATP/min. mg protein. As a marker of mitochondrial efficiency, the ATP/O ratio was calculated as ATP production rate/state 3 oxygen consumption ratio (Vanasco et al., 2012).

### Western Blot Analysis

Pancreas were removed and homogenized in 1 ml of ice-cold lysis buffer with 50 mM Hepes, 100 mM NaCl, 1 mM EDTA, 20 mM NaF, 20 mM Na_4_P_2_O_7_, 1 mM NaVO_3_, 1% Triton x-100, 1% SDS, pH 7.4; plus the addition of a mix of proteases inhibitors (1 μg/ml peptatin, 1 μg/ml aprotinin, 1 μg/ml leupeptin, and 0.4 mM phenylmethanesulfonyl fluoride). After an incubation for 10 min at 2°C, the sample was sonicated twice (30 s with 1 min interval) and centrifuged at 800 *g* for 20 min. The supernatant was then used for the western blot analysis (Towbin et al., 1979).

Equal amounts of proteins (80 μg) were separated by SDS-PAGE (7.5, 10, or 12%) and blotted into nitrocellulose films. Non-specific binding was blocked by incubation of the membranes with 5% nonfat dry milk in PBS for 1 h at room temperature. Membranes were incubated with the corresponding primary antibody at a dilution of 1:500 overnight at 4°C (Towbin et al., 1979). After incubation, nitrocellulose membrane was washed three times with PBS-Tween and then incubated with the respective secondary antibody conjugated with horseradish peroxidase (dilution between 1:10,000 and 1:5,000) for 60 min under continuous agitation. Membranes were revealed with ECL reagent. Band images were quantified by the ImageJ Software. Results were expressed as relative to β-actin/β-tubulin expression.

### Processing of Samples for Analysis by Electron Microscopy and Micrograph Analysis

Animals were euthanized in a CO_2_ chamber and pancreas was rapidly removed and washed with 0.1 M K_2_HPO_4_/KH_2_PO_4_, pH 7.4, and cut into cube of 1 mm^3^. Tissue sample was fixed with 2.5% glutaraldehyde in 0.1 M K_2_HPO_4_/KH_2_PO_4_ (pH 7.4) for 2 h, and post-fixed in 1% osmium tetroxide in 0.1 M K_2_HPO_4_/KH_2_PO_4_ at 0°C for 90 min. Afterwards, samples were contrasted with 5% uranyl acetate at 0°C for 2 h, dehydrated, and embedded in Durcupan resin (Fluka AG, Switzerland) at 60°C for 72 h. Ultrathin sections were cut and observed with a Zeiss EM 109 transmission electron microscope (Oberkochen, Germany) and representative digital images were captured using a CCD GATAN ES1000W camera (CA, United States). Damaged mitochondria and mitochondria with swelling were analyzed.

### Cells Culture, Differentiation, Treatment, and Transfection

AR42J pancreatic acinar cells were grown in a high glucose DMEM medium GlutaMAX^TM^ supplement, supplemented with 10% fetal bovine serum and 100 μg/ml streptomycin. Cells were differentiated using 100 nM dexamethasone for 48 h.

In order to develop a model for studying the direct effect of hyperstimulation of CCK-R, differentiated AR42J cells were treated with 7.4 μM caerulein at different time intervals (from 0 to 60 min) (Grasso et al., 2011). Polyethylenimine (PEI) transfection Reagent (Sigma) was used to perform the transfection of AR42J cells with different plasmids: (a) RFP-LC3 plasmid which codes for the LC3 protein, involved in autophagy (Ropolo et al., 2007), (b) GFP-VMP1 plasmid (Dusetti et al., 2002), which codes to VMP1 protein involved in selective autophagy (Grasso et al., 2011), (c) pcDNA4/V5-His-rVMP1 expression plasmid (Ropolo et al., 2007), which encodes VMP1 protein and V5 as carboxyl-terminal tag, (d) pMITO, a tandem-tagged RFP-EGFP chimeric plasmid pAT016 encoding a mitochondrial targeting signal sequence fused in-frame with RFP and EGFP genes, as a mitophagy reporter plasmid (GFP-RFP-pMITO) (Kim et al., 2013; Ojeda et al., 2018). This plasmid loses the green color at acidic pH, which makes a sophisticated marker of mitochondrial input to the autolysosome, that is, a good marker of mitophagy (Kim et al., 2013). (e) GFP-sh-VMP1 plasmid for VMP1 down-regulation designed by Ropolo et al. (2020).

### Confocal Microscopy

After GFP-RFP-pMITO transfection, treated AR42J cells were observed by the inverted confocal microscope Olympus FV1000 (PLAPON/1.42) to quantify mitophagy. The area of mitochondria per cell localized in autolysosomes (RFP-MITO) was quantified using the Fiji-win64 software.

To analyze mitochondria and lysosomes localization, treated AR42J cells were incubated with 200 nM of MitoTracker Red CMXRos and 50 nM of LysoTracker Blue DND-22 (Invitrogen) for 30 min at 37°C and subsequently washing them three times with PBS. Cells images were acquired through an Olympus Confocal Microscope FV1000 and images were acquired using the Zen Blue software (Zeiss) and processed on the AxioVision 4.2 software (Carl Zeiss). To analyze lysosomal area per cell, treated AR42J cells labeled with LysoTracker were analyzed in an Olympus Confocal Microscope FV1000 and quantified using the Fiji-win64 software in arbitrary units.

### Immunofluorescence

AR42J cells were fixed for 15 min with 4% p-formaldehyde in PBS and immediately washed three times with PBS. Then, cells were treated with triton X-100 0.1% in PBS for 5 min, washed three times with PBS, and incubated in FBS 10% in PBS for 60 min. Later, cells were incubated with polyclonal antibodies against Parkin1 (P6248, Sigma-Aldrich; 1:100 diluted) overnight at 4°C. Rabbit anti-mouse Alexa Fluor 590 (Molecular Probe) antibody was used for immunofluorescence. Samples were mounted in DABCO (Sigma-Aldrich). The cell images were acquired through an Olympus Confocal Microscope FV1000 with Spectral Detection System by Diffraction Network. Images were acquired using the Zen Blue software (Zeiss) and processed on the AxioVision 4.2 software (Carl Zeiss).

### Immunoisolation of Autophagosomes

Autophagosomes were immuno-isolated using magnetic beads fused to anti-VMP1 or anti-V5 monoclonal antibodies, according to the previously described method (Ropolo et al., 2019). The presence of VDAC, as a mitochondrial marker, was evaluated by Western blot in autophagosome fractions isolated from caerulein-treated AR42J cells transfected with pcDNA4/V5-His-rVMP1.

### Mitochondrial Membrane Potential Gradient and Mitochondrial Mass Assay in AR42J Cells

AR42J were incubated in the dark at 37°C and 5% of CO_2_ for 15 min in 500 nM of tetramethylrhodamine methyl ester (TMRM, Invitrogen, California, United States), a potentiometric cationic probe red-orange-fluorescent dye that is permeable in active mitochondria with intact membrane potential, or with 100 nM MitoTraker^TM^ Deep Red (MTDR, Invitrogen, CA, United States) that reflexes mitochondrial mass. After the incubation, cells were acquired by a FACSCanto (BDBiosciences) equipped with a 488 and 640 nm argon laser. To exclude debris, samples were gated based on light-scattering properties, and 30,000 events per sample were collected. Emission of TMRM and MTDR were measured in the PE and APC channel and the mitochondrial depolarization and mass were calculated by measuring the decrease or increase in the fluorescence intensity ratio, respectively. Autofluorescence was evaluated in the samples without probe. Total depolarization induced by 2 μM m-CCCP was used as a positive control.

### Statistics

Results were expressed as mean values ± SEM and represent the mean of, at least, five independent experiments. ANOVA followed by the Dunnett test was used to analyze differences among experimental groups. When two variables were analyzed, a Two-way ANOVA followed by the Tukey test was used. Statistical significance was considered at *p* < 0.05.

## Results

### *In vivo* Model of AP

#### Mitochondrial Function Is Early Affected in the Rat Model of AP

**FIGURE 1:**
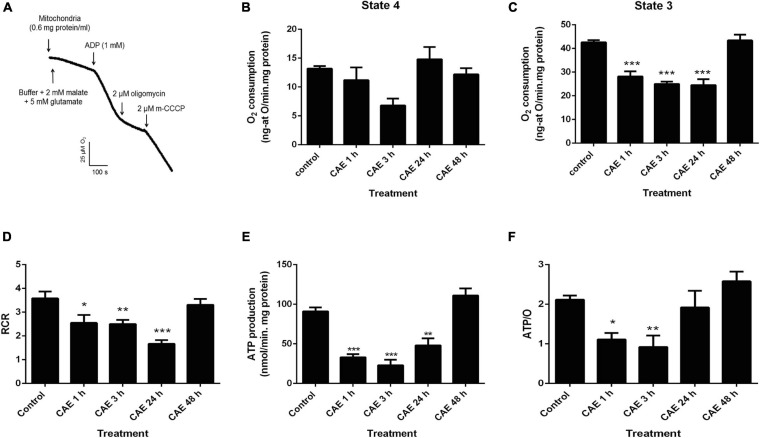
Experimental pancreatitis produces mitochondrial dysfunction in rats. **(A)** Representative traces obtained during the assessment of pancreatic mitochondrial O_2_ consumption in rest (state 4) and active (state 3) metabolic state, and after the addition of 2 μM oligomycin (state 4o) and 2 μM m-CCCP (state 3u) to the reaction chamber, in the control animal. Pancreatic mitochondria respiration in the control and CAE-treated animals in state 4 **(B)** and state 3 **(C),** ***p < 0.001 with respect to control group by ANOVA-Dunnett test, *n* = 5**. (D)** As a marker of mitochondrial coupling, respiratory control rate (RCR) was calculated as state 4/state 3. *p < 0.05 with respect to the control group by ANOVA-Dunnett test, *n* = 5; **p < 0.01 with respect to the control group by ANOVA-Dunnett test, *n* = 5; ***p < 0.001 with respect to the control group by ANOVA-Dunnett test, *n* = 5. **(E)** ATP production rate in pancreatic mitochondria from the control and CAE-treated rats. Malate plus glutamate were used as substrates. **p < 0.01 with respect to the control group by ANOVA-Dunnett test, *n* = 5; ***p < 0.001 as compared with the control group, ANOVA-Dunnett test. **(F)**. As a marker of mitochondrial efficiency, ATP/O rate was calculated as ATP production rate/state3 oxygen consumption ratio, *p < 0.05 with respect to the control group by ANOVA-Dunnett test, *n* = 5; **p < 0.01 with respect to the control group by ANOVA-Dunnett test, *n* = 5.

With the aim of analyzing pancreatic mitochondrial function during mild pancreatitis, two different approaches were used in the animal model: O_2_ consumption and ATP production rates which were measured in the isolated mitochondria. Figure 1A shows a representative trace of mitochondrial O_2_ consumption in control conditions. The first slope represents the O_2_ consumption corresponding to the mitochondrial resting respiration (state 4) in the presence of substrates, and the second slope represents the maximal physiological rate of O_2_ uptake (state 3) in the presence of substrates plus ADP. Figures 1B,C show the results obtained in rats with AP. While no significant differences between experimental pancreatitis and controls were found in resting respiration (Figure 1B), a significant decrease of O_2_ consumption in state 3 was found during the first 24 h of experimental pancreatitis (CG: 40 ± 5 ng-atO/min.mg protein, *p* < 0.01) (Figure 1C). Respiratory control ratio (RCR), calculated as the ratio between state 3/state 4 respiration rates, are shown in Figure 1D. RCR was found significantly decreased in experimental pancreatitis, without recovering after 24 h of the first CAE injection (Figure 1D), evidencing uncoupling of the mitochondrial respiratory chain from ATP production. Interestingly, RCR returned to control values after 48 h. Figure 1E shows a significant decrease (40–60%) of mitochondrial ATP production rates in the experimental model compared to controls (control value: 86.9 ± 0.9 nmol ATP/min mg protein, *p* < 0.05). These mitochondrial parameters spontaneously recovered by 48 h post the first CAE injection. To analyze mitochondrial efficiency, ATP/O ratio was measured, and data are shown in Figure 1F. ATP/O ratio was significantly reduced during the first 3 h after the CAE first injection, while it was completely recovered to control values at 24 h of treatment. These findings show that mitochondrial function is significantly affected during the acute phase of the experimental pancreatitis, but it is recovered after 48 h suggesting the induction of mitochondrial restoration mechanisms.

#### Mitochondrial Dynamics Changes Induced by Experimental AP

**FIGURE 2:**
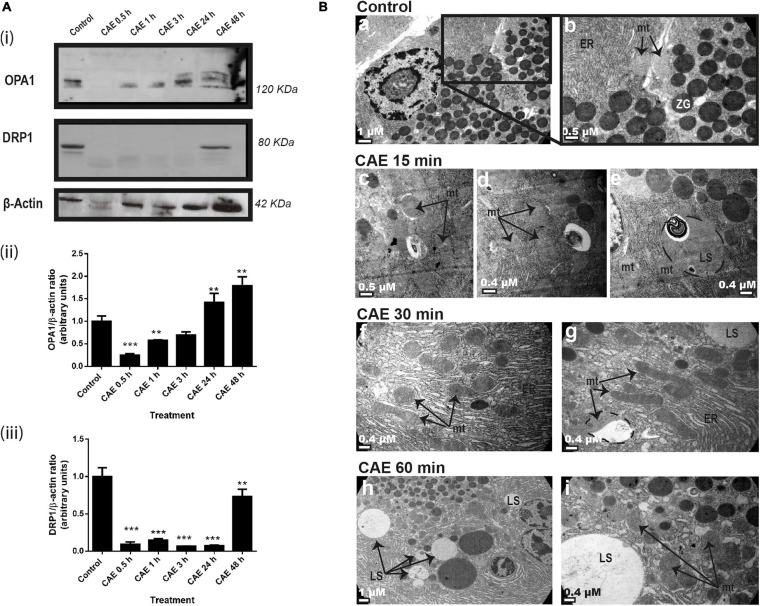
Mitochondrial dynamics during experimental AP in rats. **(A)** Panel **(i)** shows typical examples of Western blots of pancreatic homogenates samples. ß-Actin was used as loading control. Bars in panel **(ii)** represent densitometric analysis of OPA1/ß-actin ratio. Bars in panel **(iii)** represent densitometric analysis of DRP1/ß-actin ratio blot measurements. **p < 0.01 as compared with the control group by ANOVA-Dunnett test; ***p < 0.001 as compared with the control group by ANOVA-Dunnett test, *n* = 4. **(B)** Representative transmission electron micrographs shows the ultrastructure of acinar pancreatic tissue. A typical normal appearance of cytosolic acinar cell, with mitochondria (mt) and zymogens granules (ZG), included in a compact and defined area of endoplasmic reticulum (ER) is observed in control animals **(a)**. No lysosomal structures are observed in this tissue **(a,b)**. The ultrastructure corresponding to animals treated with CAE **(b–i)** showed a disorganization and swelling of endoplasmic reticulum (compatible with reticulum stress) and it is evident that an increase in lysosomal structures is a time dependent manner **(b–i)**. Scale bars: 1 μm in panels **(a,h)**; 0.5 μm in panels **(b,c)**, 0.4 μm in panels **(d,e,f,g,i)**.

In order to evaluate mitochondrial dynamics, the expression of OPA1 (a marker of mitochondrial fusion) and DRP1 (a marker of mitochondrial fission) were analyzed using Western blot as shown in Figure 2A. Figure 2Ai shows a dramatic reduction of DRP1 being undetectable after 30 min of the first injection of CAE and remaining undetectable at 24 h. OPA1 expression was significantly decreased after 30 min of pancreatitis (Figure 2Ai). After 3 h of the first injection of CAE, OPA1 values returned to basal values. Although DRP1 suddenly increased, it remained lower than basal values (Figure 2Ai), probably due to the regulation of the sustained OPA1-dependent mitochondrial fusion effect. Western blots quantification is shown in Figures 2Aii,iii. Taking into account that we found significant changes in the expression of mitochondrial dynamics proteins after 30 min of CAE treatment, we analyzed ultrastructural changes in pancreas tissue during the early stage of AP. Figure 2B shows the electron microscopy ultrastructural analysis of pancreas tissue from the control (Figure 2B, panels a and b) and from animals subjected to experimental AP (Figure 2B, panels c to i). In control animals, pancreatic acinar cells cytosol appears normal, with zymogen granules and mitochondria embedded in a defined and compact endoplasmic reticulum area, and no lysosomes are observed. On the contrary, characteristics that resemble endoplasmic reticulum stress and mitochondrial dynamics were observed in animals with AP after CAE treatment, accompanied with an increase in the amount and size of lysosomal structures (Figure 2B, panels c to i). These results demonstrate a dysregulation of mitochondrial dynamics during the development of AP, accompanied by endoplasmic reticulum stress and increased lysosomal activity.

#### Mitophagy Is Induced in the Rat Model of AP

**FIGURE 3:**
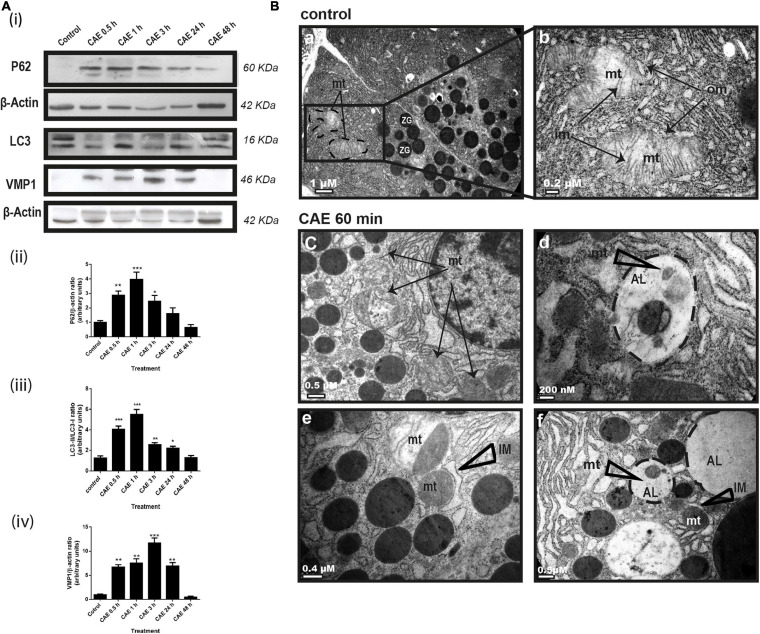
Experimental pancreatitis induces autophagy and mitophagy in rats. **(A)** Changes in mitochondrial autophagy proteins expression during experimental AP. Panel **(i)** shows typical Western blots of pancreatic homogenates samples. ß-Actin was used as loading control. Bars in panel **(ii)** represent densitometric analysis of PG62/ß-actin ratio. Bars in panel **(iii)** represent densitometric analysis of LC3-II/LC3-I ratio blot measurements. Bars in panel **(iv)** represent densitometric analysis of VMP1/ß-actin ratio. *p < 0.05 as compared with the control group, ANOVA-Dunnett test; **p < 0.01 as compared with the control group, ANOVA-Dunnett test; ***p < 0.001 as compared with the control group, ANOVA-Dunnett test; *n* = 4. **(B)** Representative transmission electron micrographs of pancreas from control **(a,b)** and CAE 60 min treated animals **(c–f)**. **(a,b)**: control animals displayed normal mitochondria morphology. Typical organization of mitochondria (mt) clearly displayed the inner membrane (im) comprizing of cristae and well defined mitochondrial outer membrane (om). **(c–f)**: 1 h after CAE administration, mitochondria displayed several abnormalities, such as loss and/or disruption of cristae, clearer matrix, and swelling [black arrows, in panels **(c,e)**]. On the other hand, “isolation membranes” (IM) can be observed that partially surround mitochondria **(e,f)** and visible autolysosomes (AL) with remains of mitochondrial structures in their interior **(d,f)**, both structures compatible with mitophagy. Scale bars: 1 μm in panel **(a)**; 0.2 μm in panel **(b)**; 0.5 μm in panels **(c,f)**; 200 nm in panel **(d)**; and 0.4 μm in panel **(e)**.

We evaluated the autophagy process in the animal model in order to have the temporal relationship between mitochondrial function and dynamics. Autophagic flux was evaluated by analyzing protein expression of p62, LC3, and VMP1. Figure 3A shows a significant increase in LC3-II expression and a significant decrease in p62 expression in AP. These results were accompanied by an increase in VMP1 expression, which is compatible with the occurrence of VMP1-dependent autophagy (Ropolo et al., 2007). Western blots quantification is shown in Figure 3A, panels i to iii. Changes in the mitochondrial ultrastructure and its relationship with the lysosome during the occurrence of AP were evaluated. In Figure 3B, panels a and b, representative electron microscopy of control pancreatic tissue is shown. Characteristic lamellar shape of acinar cell mitochondria can be observed. The conserved mitochondrial ultrastructure can be clearly distinguished, given by the integrity of the outer and the internal membranes which form the mitochondrial crests. However, in animals treated with CAE for 60 min, mitochondrial swelling (evidenced by its roundness, clearance of the matrix, separation and disruption of mitochondrial crests) were observed (Figure 3B, panels c and e). In addition, structurally polarized mitochondria were observed; characteristically divided by intramitochondrial septa that separates the deeply damaged from normal mitochondrial portions (Figure 3B, panels c and e). Moreover, presence of autophagic vesicles with engulfed mitochondria (Figure 3B, panels d and f), and “isolation membrane” structures that partially surround mitochondria were found in the pancreas from the experimental model after 60 min of pancreatitis (Figure 3B, panels e and f). After 48 h of the first CAE injection, mitochondrial morphology has no differences with respect to the normal pancreas. These data are consistent with the occurrence of mitophagy of damaged mitochondria during the acute phase of experimental mild pancreatitis in rats.

### *In vitro* Model of AP

Using an *in vitro* model previously described by the authors (Grasso et al., 2011), we defined the molecular mechanism involved in mitophagy of damaged mitochondria during pancreatitis next. For this goal, we have evaluated mitochondrial function, dynamics, and mitophagy at the cellular level in AR42J pancreatic acinar cells under hyperstimulation of the G-coupled receptor of CCK, a model for early cellular events in AP (Grasso et al., 2011).

#### Mitochondrial Dynamics Induced by CCK-R Hyperstimulation in AR42J Cells

**FIGURE 4:**
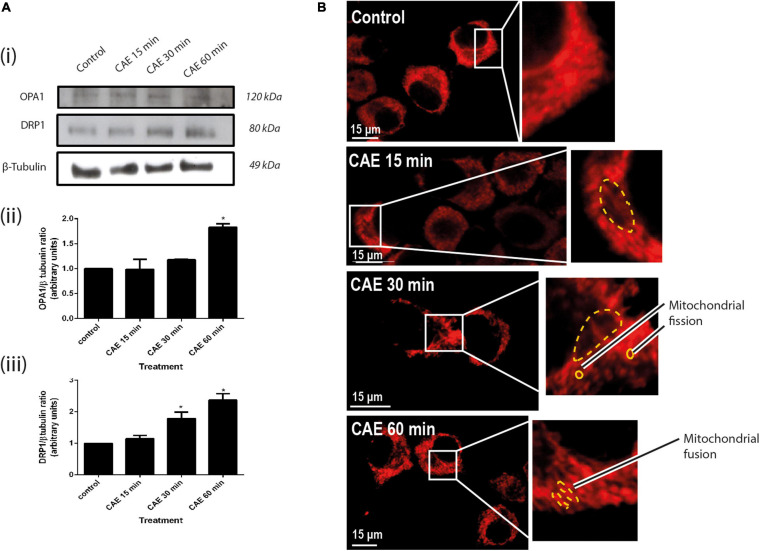
Mitochondrial dynamics in AR42J cells under CCK-R hyperstimulation. **(A)** OPA1 and DRP 1 protein expression of AR42J pancreatic acinar cells treated with vehicle and CAE. Panel **(i)** shows typical examples of Western blots of pancreatic homogenates samples. ß-tubulin was used as loading control. Bars in panel **(ii)** represent densitometric analysis of OPA1/ß-tubulin ratio. Bars in panel **(iii)** represent densitometric analysis of DRP1/ß-tubulin ratio blot measurements. *p < 0.05 as compared with control group, ANOVA-Dunnett test, *n* = 4. **(B)** Red-MitoTracker probe was used to mark the mitochondrial population. Control, CAE 15 min, CAE 30 min, and CAE 60 min representative images are shown. Populations of shortened mitochondria [panel **(B)**, CAE 30 min] and elongated mitochondria [panel **(B)**, CAE 60 min], morphologies compatible with changes in mitochondrial dynamics (mitochondrial fission and fusion), are observed in cells treated with CAE. Scale bar represents 15 μm.

Given that mitochondrial dysfunction can lead to changes in mitochondrial dynamics (Ferree and Shirihai, 2012), markers of mitochondrial dynamics such as the expression of DRP1 (mitochondrial fission) and OPA1 (mitochondrial fusion), as markers of mitochondrial dynamics, as well as the mitochondrial morphology, were evaluated. Figure 4Ai shows representative DRP1 and OPA1 Western blots. An earlier increase of DRP1 expression was found after 30 min, while OPA1 increased after 60 min of CAE treatment (Figure 4A, panels ii and iii). Consistent with these results, morphological images using the MitoTracker probe showed two mitochondrial populations: a small and rounded mitochondrial population after 30 min of treatment and a marked elongated mitochondrial population after 60 min of treatment (Figure 4B). As a whole, these results support the occurrence of mitochondrial fission after 30 min accompanied by mitochondrial elongation after 60 min of CCK-R hyperstimulation.

#### Mitophagy Is Induced by CCK-R Hyperstimulation in AR42J Cells

**FIGURE 5:**
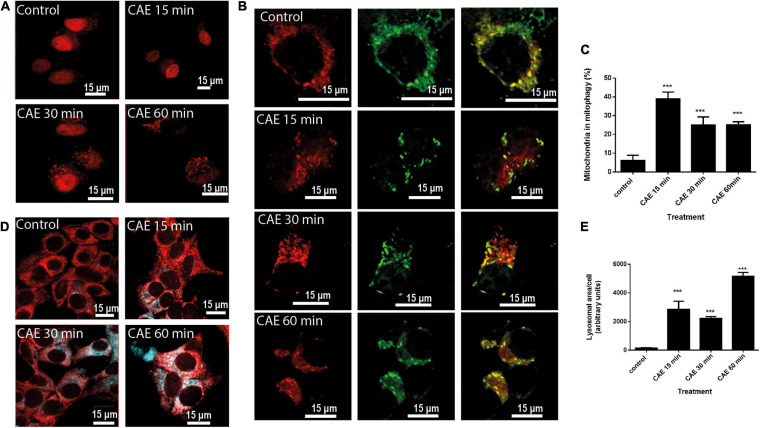
Mitophagy is induced in AR42J cells under CCK-R hyperstimulation. **(A)** Representative confocal microscopy images of AR42J cells transfected with plasmid encoding for RFP-LC3 (control, CAE 15 min, CAE 30 min, and CAE 60 min). **(B)** AR42J cells treated with CAE and transfected with plasmids encoding for RFP-GFP-pMITO. The GFP signal is quenched at the lower pH of lysosomes, while RFP can be consistently visualized. Yellow fluorescence (RFP merged with GFP) indicates normal mitochondria population, whereas red fluorescence (RFP) indicates population of mitochondria undergoing mitophagy. **(C)** Quantification of the percentage of the mitochondrial population found in mitophagy. ***p < 0.001 compared with the control group, ANOVA-Dunnett test. **(D)** Representative confocal micrographs showing mitochondria detected with Red-MitoTracker and lysosome detected with Blue-LysoTracker in treated pancreatic AR42J cells (control, CAE 15 min, CAE 30 min, and CAE 60 min). Scale bar represents 15 μm. **(E)** Lysosomal area quantification per cell (in arbitrary units). ***p < 0.001 compared with the control group, ANOVA-Dunnett test.

We evaluated autophagy in cells transfected with RFP-LC3 as an autophagy marker. Figure 5A shows a time-dependent increase in autophagosome formation after CCK-R hyperstimulation evidenced by LC3 recruitment. In order to identify mitophagy, AR42J cells treated with CAE were transfected with the specific tandem probe pMITO-RFP-GFP. Using confocal analysis (Figure 5B), control cells exhibited a mitochondrial population mostly labeled in yellow indicating healthy organelles. On the contrary, two separate mitochondrial populations were observed after CCK-R hyperstimulation. One of them remained labeled in yellow, and the other one located in the apical area of the cytoplasm, labeled in red, indicating their autolysosome location. Therefore, confocal analysis using the specific tandem probe confirmed the occurrence of mitophagy in the cell model of AP. Figure 5C shows that mitophagy is significantly increased under hyperstimulation of CCK-R.

Then, the subcellular distribution of healthy mitochondria (labeled with Red-MitoTracker) and lysosomes (labeled with blue-LysoTracker) was evaluated. Figure 5D shows healthy mitochondria labeled in red that did not localize with tenuous and small lysosomes appearing in the basal area of the control cells. While, under CCK-R hyperstimulation, the presence of large lysosomes was clearly evident (Figure 5D). Lysosomal area per cell was quantified and it is shown in Figure 5E. This “lysosomal pocket” coincided with the apical area where mitophagy was observed in Figure 5B, evidencing the presence of autolysosomes with damaged and degraded mitochondria inside that were not able to be labeled with the MitoTracker.

#### CCK-R-Hyperstimulation Induced Mitochondrial Dysfunction and Mitophagy in Pancreatic Acinar Cells

**FIGURE 6:**
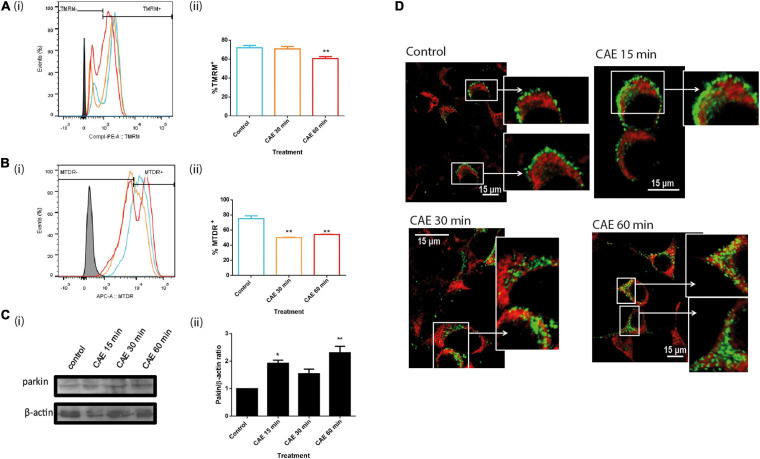
Mitochondrial depolarization induces parkin-dependent mitophagy during CCK-R hyperstimulation. **(A)** Mitochondrial inner membrane potential evaluated by flow cytometry using TMRM probe **(i)** Overlaid histograms of mitochondrial event versus TMRM fluorescence intensity. Control (light blue), CAE 30 min (orange), CAE 60 min (red), and autofluorescence (full black). **(ii)** % TMRM^+^ cells quantification. **p < 0.01 compared with the control group, ANOVA-Dunnett test. **(B)** Mitochondrial mass evaluated by flow cytometry used MTDR. **(i)** Overlaid histograms of mitochondrial events versus MTDR fluorescence intensity. Control (light blue), CAE 30 min (orange), CAE 60 min (red), and autofluorescence (full black). **(ii)** % MTDR^+^ cells quantification. **p < 0.01 compared with the control group, ANOVA-Dunnett test. **(C)** Parkin expression by Western Blot assay in AR42J cells treated with CAE at different times. Panel **(i)** shows typical Western Blots of pancreatic homogenates samples. ß-actin was used as the loading control. Bars in panel **(ii)** represent densitometric analysis of parkin/ß-actin ratio. *p < 0.05 as compared with the control group, ANOVA-Dunnett test, *n* = 4. **(D)** Representative confocal microscopy images of AR42J cells treated with CAE showing intracellular changes distribution of mitochondria (detected with Red-MitoTracker probe) and Parkin (detected by immunofluorescence in green).

To determine mitochondrial function in AR42J cells that were submitted to CCK-R hyperstimulation, mitochondrial inner membrane potential was analyzed by flow cytometry using the TMRM potentiometric probe. Figure 6Ai shows representative time-dependent course histograms of hyperstimulation as a function of TMRM fluorescence intensity; panel Aii shows histograms quantification, represented as the percent of AR42J cells that preserve the inner membrane mitochondrial potential. In Figure 6Aii, a significant decrease in mitochondrial internal membrane potential was observed after 60 min of CCK-R hyperstimulation, indicating that mitochondrial damage in this cellular model of pancreatitis. Besides, to estimate the mitochondrial degradation in pancreatic cells submitted to CCK-R-hyperstimulation, an MTDR probe that remains inside intact mitochondria was used. Figure 6B is a representative histogram showing a time-dependent variation in the mitochondrial mass as a function of MTDR fluorescence intensity showing a significant decrease in MTDR fluorescence after 30 and 60 min of CAE hyperstimulation. These data suggest that pancreatitis not only affects mitochondrial function but also induces early and significant degradation of damaged mitochondria as well.

Together with Mnf2, Parkin1 is one of the proteins that associates to the external mitochondrial membrane when mitochondrial membrane potential decreases. Parkin1 is a ubiquitin ligase that labels proteins of mitochondrial outer membrane to serve as substrates of autophagy-cargo recognition molecules such as p62, which interacts with LC3 to initiate selective autophagy. Figure 6C shows that under CCK-R hyperstimulation, Parkin1 expression assayed by western blot showed a rapid and significant increase under CCK-R hyperstimulation. Moreover, Figure 6D shows that while Parkin1 labeling is light and located in the cytosol in control cells, a marked translocation of Parkin1 from cytosol to mitochondria was observed after CCK-R hyperstimulation. These findings suggest that during AP, Parkin1 recognizes and recruits to damaged mitochondria in order to label them for autophagic degradation.

#### VMP1-Mediated Mitochondrial Degradation Is Induced in Pancreatic Acinar Cells Under CCK-R-Hyperstimulation

**FIGURE 7:**
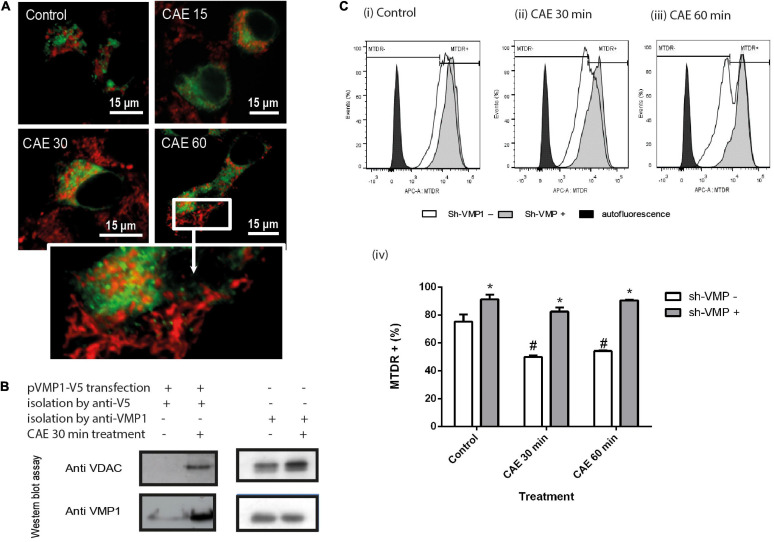
Identification of the novel VMP1 pathway mediating selective mitophagy in experimental pancreatitis. **(A)** AR42J transfected cells with plasmid encoding for GFP-VMP1 and treated with CAE. Red-MitoTracker was used to show mitochondrial population. **(B)** Western Blot of isolated autophagosomes by magnetic beads linked to anti V5 or VMP1 antibodies from AR42J CAE-treated cells. The presence of the mitochondrial protein VDAC is observed only in the cells treated with CAE. **(C)** VMP1 inhibition attenuates mitophagy during pancreatitis, evaluated by flow cytometry using MTDR. AR42J cells were transfected with GFP-shVMP1 plasmid 48 h before CAE treatment. Overlaid histograms of AR42J events versus MTDR fluorescence intensity. shVMP1-transfected population and not transfected in panel **(i)** control, **(ii)** CAE 30 min, and **(iii)** CAE 60 min. **(iv)** MTDR fluorescence quantification, *p < 0.01 as compared to the same treatment without transfection; ^#^*p* < 0.01 as compared to the control group Two-way ANOVA-Tukey test.

To investigate if VMP1 is involved in the selective autophagic degradation of damaged mitochondria during pancreatitis, the cells were transfected with EGFP-VMP1 and labeled mitochondria with red-MitoTracker. Figure 7A shows that in control cells, healthy mitochondria were labeled in red and did not localize with green VMP1. On the contrary, a dramatic redistribution of VMP1, now surrounding rounded mitochondria, can be observed after CCK-R hyperstimulation. Taking into account that VMP1 is a transmembrane protein of the autophagosomes, we isolated autophagosomes using magnetic beads fused to anti-VMP1 antibody, or anti-V5 antibody from AR42J cells expressing VMP1-V5. Both methods showed that after CCK-R hyperstimulation, autophagosome fractions contained mitochondrial VDAC markers. These data further confirm that VMP1 is involved in mitophagy during AP (Figure 7B). To investigate if VMP1 is required for mitochondrial degradation during pancreatitis, VMP1 expression was downregulated using a dual sh-VMP1 plasmid labeled with GFP allowing to distinguish two cellular outcomes after transfection and exclude by gating GFP negative cells. GFP positive cells were analyzed by flow cytometry to estimate mitochondrial degradation during experimental pancreatitis. As shown in Figure 7C, an increase in the MTDR^+^ population was observed in all cells transfected with sh-VMP1 plasmid compared to non-transfected cells, for each treatment time. Furthermore, no significant changes were observed between the different treatment times in sh-VMP1 transfected cells. These results suggest that VMP1 inhibition preserves mitochondrial mass by inhibiting mitophagy. Together, these results show that VMP1 mediates the selective autophagy of damaged mitochondria during experimental pancreatitis. The fact that mitochondrial mass is preserved, even in control cells, suggests that VMP1-mediated mitophagy might also function as a homeostatic process regulating mitochondria function in pancreatic acinar cells.

## Discussion

AP is an inflammatory disease for which its pathogenesis is poorly understood and lacks a specific treatment. However, most of the cases are mild and self-limited forms and pancreas morphology and physiology is totally recovered. During the development of AP, pancreatic intracellular trypsinogen activation, mitochondrial damage with the consequent ATP depletion and inflammatory response are characteristic pathophysiological features (Maléth et al., 2013). This work is focused on the comprehensive study of mitophagy, its molecular mechanisms and its relationship with mitochondrial dynamics in two experimental models of AP. We have analyzed mitochondrial fusion and fission, as well as mitophagy, using two models: an *in vitro* cellular model, and *in vivo* model of mild and self-limited form of AP. These processes, which were historically described separately, lead to structural morphological changes necessary to maintain or restore mitochondrial function under physiological and pathological conditions.

While OPA1 is the protein related to mitochondrial fusion and elongation, DRP1 is a key molecule in the mitochondrial fission, a mechanism considered necessary to obtain mitochondrial fragments that are sequestered and degraded by autophagy (Gomes et al., 2011). Li et al. (2019) shows that DRP1 nitration (due to the increase of the powerful oxidant ONOO-) promotes its assembly and its recruitment to the mitochondrial outer membrane and induces the process of mitophagy mediated by the Pink1/Parkin1 pathway. This mechanism is relevant in the cell response to human pathologies. For instance, deficiency of OPA1 expression is accompanied by an excessive increase in fragmented mitochondria and a dysregulated mitophagy in mitochondrial optic neuropathies (Liao et al., 2017). Therefore, changes in DRP1 and OPA1 expression are not only involved in mitochondrial dynamics but also in the clearance of damaged mitochondria by autophagy (Jin and Youle, 2012). In the present study, using the *in vivo* model of AP in rats, we found mitochondrial damage along with altered mitochondrial dynamics and autophagic degradation of mitochondria. These features were evidenced by the 24 h time course changes in DRP1 and OPA1 values and the simultaneous increase in autophagic markers such as LC3 and p62, as well as high expression of the selective autophagy marker VMP1 (Grasso et al., 2011). Mitochondrial dynamics changes were confirmed by electron microscopy, which showed ultrastructural damage such as polarized (fission) and elongated (fusion) mitochondria. Furthermore, significant increases in lysosomal structures were observed with internalized mitochondrial structures in degradation processes. Interestingly, the expression of proteins involved in fusion, fission, and autophagy returned to control values after 48 h, along with functional recovery of mitochondria.

In order to understand the molecular pathway of the selective degradation of damaged mitochondria in pancreatitis, we studied mitophagy in pancreatic cells under CCK-R hyperstimulation. Using this *in vitro* model, we observed an increase in Parkin1 expression, previously reported in AP by Biczo et al. (2018). Moreover, we observed the redistribution of Parkin1 from a cytosolic location to damaged mitochondria areas. Also, the increment of large lysosomes containing degraded mitochondria are in agreement with the described mitophagy. Using the GFP-RFP-pMITO tandem probe, we demonstrated that mitophagy is early induced in the pancreatic cell model under CCK-R hyperstimulation and remains activated during 60 min. Interestingly, two mitochondrial populations morphologically distinguishable were observed simultaneously. Those polarized after fission of damaged mitochondria, and the elongated ones after fusion of the healthy mitochondria, suggesting a selective mechanism of degradation of damaged mitochondria allowing the recovery of energetic status. Pink1 is a protein located in the mitochondrial outer membrane, where it is continuously degraded by various processes (Bingol and Sheng, 2016). However, mitochondrial depolarization or malformed proteins accumulation in its matrix (both signs of mitochondrial damage) trigger mechanisms that stabilize Pink1 with its consequent accumulation in mitochondria (Geisler et al., 2010; Vives-Bauza et al., 2010). This Pink1 accumulation is Parkin’s recruitment signal to the outer mitochondrial membrane, a signal recognized by the autophagic machinery for the degradation of these damaged mitochondria. Moreover, for the first time, here we are reporting the VMP1 involvement in the mitophagy process during AP, observable not only by repositioning around damaged mitochondrial populations, but also by the detection of mitochondria in autophagosomes specifically isolated with anti-VMP1 antibodies. Downregulation of VMP1 avoided mitochondrial degradation and confirmed that VMP1 expression is required for mitophagy during AP. We present evidence of a novel DRP1-Parkin1-VMP1 pathway, which mediates the selective degradation of damaged mitochondria by mitophagy in AP.

Furthermore, our results confirmed the early decrease of mitochondrial function in the mild model of pancreatitis through the determination of mitochondrial ATP production rate and the mitochondrial O_2_ consumption and ATP/O ratio, as it was previously reported (Mukherjee et al., 2016). Mitochondrial dysfunction in pancreatitis has been described through decreased mitochondrial membrane potential, increased calcium uptake; decreased ATP levels, considering the latter as a marker of ATP synthase activity; or indirect determinations of ATP synthase activity in submitochondrial particles (Biczo et al., 2018). To our knowledge, direct assessment of the ATP production rate in intact mitochondria, as well as ATP/O ratio allows a better understanding of the degree of mitochondria dysfunction during AP.

In physiological conditions, more than 90–95% of the O_2_ consumed by living beings is reduced to H_2_O through the mitochondrial respiratory chain, by oxidative phosphorylation to produce ATP. Moreover, between 1 and 2% of the O_2_ consumed is partially reduced to O${}_{2}^{\cdot -}$ and H_2_O_2_ in the mitochondria, being the main source of active oxygen species as signaling molecules (Boveris and Cadenas, 1982). We found that both the mitochondrial O_2_ consumption rate and the mitochondrial ATP production were significantly decreased (39 and 50%, respectively) up to 24 h of experimental AP. These results together with an abrupt decrease in ATP/O rate, as a marker of mitochondrial efficiency (Vanasco et al., 2012), suggest that part of the O_2_ consumed is not used for ATP production, and it is used in other metabolic pathways such as in mitochondrial ROS generation instead. An increase in mitochondrial ROS has been observed by Booth et al. (2011b), during bile acid injury of pancreatic acinar cells. Moreover, MitoQ (an antioxidant targeted to mitochondria) reduces both inflammation and the presence of edema in acinar cells treated with caerulein (Huang et al., 2015). On the other hand, the decrease in ATP availability may lead to the inefficiency of the Ca^2+^ (ATP-dependent) pumps (Booth et al., 2011a), responsible for restoring basal Ca^2+^ cytosolic levels. In this way, apoptosis might also be compromised, since caspase activation is an ATP-dependent process, leading cells to necrosis.

The central role of mitochondrial dysfunction and impaired autophagy were reported in different animal models of severe AP such as in mice treated with L-arginine-induced pancreatitis (Biczo et al., 2018). In these models, mitochondrial dysfunction causes pancreatic ER stress, impaired autophagy, and deregulation of lipid metabolism. However, the administration of trehalose, an autophagy inductor, prevents intracellular trypsinogen activation, necrosis, and other parameters of pancreatic injury (Biczo et al., 2018). Also, we previously demonstrated that the induction of autophagy in the pancreas by the transgenic expression of VMP1 does not only induce pancreatitis but also prevents intracellular trypsinogen activation, necrosis, and other parameters of pancreatic injury in the mouse caerulein model of pancreatitis (Grasso et al., 2011). Interestingly, in the rat model of mild pancreatitis, we showed that the mitochondrial function is spontaneously and completely restored when pancreas tissue is also recovered, after 48 h of caerulein treatment. These findings suggest that the acinar cell can set up molecular mechanisms to restore cellular bioenergetics leading to the pancreas recovering.

**FIGURE 8:**
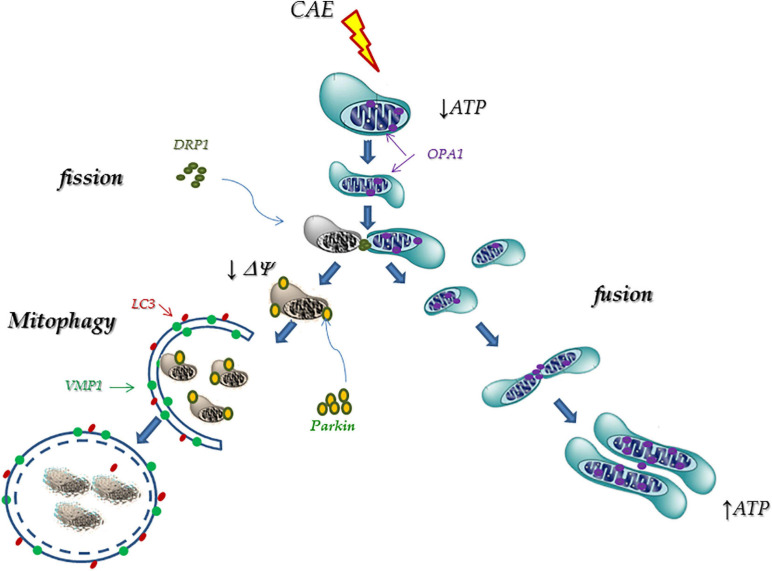
Proposed mechanism: Mitophagy as a cellular rescue mechanism during pancreatitis. During AP, mitochondrial failure is able to induce phenotypic changes in acinar cells (OPA1, DRP1, Parkin1, and VMP1 expressions) that triggers mitochondrial remodeling processes. These changes include fusion events (through OPA1) which allow internal rearrangement of their structure; and fission events (through DRP1) that originate new functional mitochondria as well as damaged and depolarized mitochondria. The latter are labeled by Parkin1, and through the VMP1-dependent autophagic pathway, are selectively detected and degraded by mitophagy within the lysosomes.

Taking our results together, we hypothesize that during AP, mitochondrial failure can induce phenotypic changes in acinar cells (OPA1, DRP1, Parkin1, and VMP1 expressions) that triggers mitochondrial remodeling processes. These changes include fusion events (through OPA1) which allow internal rearrangement of their structure; and fission events (through DRP1) that originate new functional mitochondria as well as damaged and depolarized mitochondria. The latter, labeled by Parkin1 and through the VMP1-dependent autophagic pathway, is selectively detected and degraded by mitophagy within the lysosomes (Figure 8). A better understanding of the molecular mechanisms involved to restore mitochondrial function, such as changes in mitochondrial dynamics and mitophagy, could be relevant in the development of therapeutic strategies in AP.

## Data Availability Statement

The raw data supporting the conclusions of this article will be made available by the authors, without undue reservation.

## Ethics Statement

The animal study was reviewed and approved by Comité Institucional para el Cuidado y Uso de Animales de Laboratorio (CICUAL-FFyB).

## Author Contributions

VV performed most of the experiments and wrote the manuscript. VV and AR designed the experiments and analyzed the results. DG, DO, MG, and TV contributed to the development of the experimental models, sample preparations, and assessments. TO performed expression plasmid designs and constructions. JQ discussed the results and revised the manuscript. SA designed and analyzed the data of mitochondrial function experiments on animal and cellular models and edited the manuscript. MV directed the work, designed the autophagy/mitophagy experiments in animal and cellular models of pancreatitis, discussed the results, and edited the manuscript. All the authors revised the final version of the manuscript.

## Conflict of Interest

The authors declare that the research was conducted in the absence of any commercial or financial relationships that could be construed as a potential conflict of interest.

## Abbreviations

AP: acute pancreatitis
CAE: caerulein
CG: control group
DRP1: dynamin-related protein 1
FAEEs: fatty acid ethyl esters
LC3: microtubule associated protein 1 light chain 3
Mnf: Mitofusin
OPA1: optic atrophy 1
PEI: polyethylenimine
PINK1: PTEN-induced kinase 1
RCR: respiratory control ratio
ROS: reactive oxygen species
VMP1: vacuole membrane protein 1 (NM_138839).
